# Numerical and statistical analysis of aluminum deep drawing using LS-DYNA coupled with Taguchi design and response surface methodology

**DOI:** 10.1038/s41598-026-43326-w

**Published:** 2026-03-26

**Authors:** Eslam Shamso, Moataz Abd El Kafy, Noha Naeim

**Affiliations:** Production Engineering and Mechanical Design Department, Faculty of Engineering, Port-Said University, Port-Said, Egypt

**Keywords:** Deep drawing, Al materials, LS-DYNA -ANSYS, Taguchi Method, ANOVA, and, Engineering, Materials science, Mathematics and computing

## Abstract

Deep drawing is one of the most widely used sheet metal forming processes in the manufacturing industry. This study presents a finite element-based investigation of the deep drawing process of aluminum circular blanks using LS-DYNA. The objective is to assess the influence of key process parameters on formability and failure modes during low-depth cup forming. A parametric design based on the Taguchi method was adopted, incorporating three levels for each of the following parameters: punch velocity (2, 5, 8 mm/s), blank thickness (1, 1.5, 2 mm), and blank holder force (5, 10, 15 kN). The output responses included maximum forming force, maximum thinning %, effective plastic strain, Von Mises stress, and Forming Limit Diagram (FLD) values. The simulation results were analyzed using ANOVA within Response Surface Methodology (RSM) to evaluate the statistical significance and interactions among the input variables. The results indicate that punch velocity is the dominant factor controlling thinning, forming force, stress, and plastic strain, followed by blank thickness, while blank holder force has a minor effect. The developed regression models exhibited strong reliability and predictive capability, with statistical validation and close agreement between predicted and actual responses.

## Introduction

Metal forming includes a large group of manufacturing processes in which plastic deformation is used to change the shape of metal workpieces. Deformation results from the use of a tool, usually called a die in metal forming, which applies stresses that exceed the yield strength of the metal. The metal therefore deforms to take a shape determined by the geometry of the die^1^. Sheet metalworking processes are forming and cutting operations performed on metal sheets, strips, and coils. The surface area-to-volume ratio of the starting metal is high; thus, this ratio is a useful means to distinguish bulk deformation from sheet metal processes. Pressworking is the term often applied to sheet metal operations because the machines used to perform these operations are presses (presses of various types are also used in other manufacturing processes)^2^. Deep drawing is a fundamental sheet metal forming technique widely applied across various industries, including medical applications, for the production of diverse metal components. The process involves the application of tensile forces to induce plastic deformation in the material, thereby shaping it into the required geometry^3^. In practice, a sheet metal blank is radially drawn into the die cavity by the downward movement of a punch. The blank is positioned on the die, and a blank holder is applied to its upper surface to regulate material flow during forming. As the punch descends, it forces the blank into the die cavity, causing the material to conform to the die geometry^4^. To enhance structural performance while reducing weight and cost, advanced high-strength steels (AHSS) and aluminum are widely selected for deep-drawn components to achieve weight and cost reduction while satisfying established safety requirements. Nevertheless, their application is constrained by manufacturing limitations, particularly reduced formability, as they permit lower deformation levels compared with mild steel^5^.

The relevant literature on deep drawing is reviewed below, highlighting key contributions from previous studies. In this context, El Mrabti et al.^6^. conducted comprehensive LS-DYNA simulations for square aluminum and high-strength steel cups, employing Taguchi and ANOVA to quantify the contribution of multiple process parameters. Their findings highlighted the dominant role of blank thickness on thinning behavior and internal energy absorption, confirming the reliability of FEA-based parametric investigations. Similarly, Mekonnen et al.^7^ studies the fabrication of Cu–SiC–Cr–Gr composites using stir casting followed by hot extrusion to enhance mechanical properties. Numerical simulations using DEFORM-3D and Taguchi-based optimization were applied to evaluate the effects of extrusion parameters on load and damage. The results showed reduced porosity and improved strength and hardness after extrusion, accompanied by a slight reduction in electrical conductivity. Aleyna Taşkın et al.^8^. combined Taguchi-based experiments with Abaqus/Explicit simulations to investigate the deep drawing of medical containers, demonstrating that blank holder force and friction coefficient significantly affect thickness reduction and punch force. Their results showed that increasing punch and die radii improves thickness uniformity and reduces forming load. Venkateswarlu et al^9^.. investigated the effect of warm forming parameters on the deep drawing of AA7075 aluminum alloy. By varying die radius, punch speed, and temperature, the study revealed the enhanced ductility and reduced springback achievable at elevated temperatures. Using Taguchi design and ANOVA, the authors identified the optimal process window for minimal thinning and wrinkle suppression. Their conclusions are instrumental in advancing warm deep drawing processes for aerospace-grade aluminum materials. Reddy et al^10^. Focused on the warm deep drawing of aluminum alloy 2017-T4, this study implemented FEA simulations using LS-DYNA in conjunction with Taguchi optimization techniques. Parameters such as lubrication, punch radius, and holding pressure were systematically examined. The findings demonstrated how elevated temperatures reduce required forming forces while enhancing strain distribution uniformity. This work offers practical insights into reducing trial-and-error in industrial applications involving warm forming. Thanh Luyen et al.^11^. validated finite element simulations of deep drawing for SPCC steel sheets, reporting excellent agreement with experimental results. Their work emphasized the influence of blank holder force, punch radius, and drawing ratio on fracture height, further reinforcing the importance of accurate numerical modeling in predicting forming limits. These studies collectively confirm that FE simulations can effectively reduce experimental cost and trial-and-error in deep drawing design. Tahjib et al.^12^ investigated thinning behavior in incremental sheet forming of AA3003-H14 aluminum using LS-DYNA. Although these studies provide valuable insights into deformation mechanisms, their focus lies outside conventional deep drawing of low-depth aluminum cups. Shafiee Sabet et al.^13^. developed a multi-factor friction model incorporating pressure, velocity, and temperature effects, which significantly improved the accuracy of deep drawing simulations compared to constant friction models. In parallel, Yufeng Pan et al.^14^. investigated hydromechanical deep drawing using numerical simulations and RSM optimization, achieving high prediction accuracy for thinning behavior. Amr Shaaban et al.^15^. presented a combined numerical and experimental investigation of the deep drawing of low-depth products, with application to an aluminum frying pan (AL99.9%). A finite element model was developed using LS-DYNA and validated against experiments, showing minimal deviation in load and thickness predictions. The validated model was then used to analyze key process parameters, revealing that cushion stiffness and blank thickness significantly affect pressing load and thinning, while punch speed has negligible influence. El Mrabti et al.^16^. studied the use of deep drawing in various industries, focusing on process failures such as springback. Process parameters were optimized using finite element simulation, artificial neural networks, and particle swarm optimization. The results showed that this integrated approach effectively minimized springback, providing a reliable tool for optimizing highly non-linear forming processes. Besart Berisha et al.^17^. presented a systematic analysis of a deep drawing process for a component designed in SolidWorks. The part geometry and all relevant dimensions were defined in detail and the chosen material was DC03 cold-rolled steel. The study includes step-by-step theoretical calculations for center of gravity, tool tolerances, die clearances (working space), and material allowances. Numerical analyses were performed using Logopress (integrated with SolidWorks) to examine strain distribution, mesh quality, stamping (forming) force, and stripper force. The combined design, theoretical and FE workflow provides a complete framework for evaluating and optimizing deep drawing performance for the selected material. Nasri S. M. Namer et al.^18^. investigated the effect of punch nose radius on the deep drawing of 2024-T4 aluminum cups, demonstrating improved formability and thickness distribution with increasing punch radius. Hussein Zein et al.^19^. studied the deep drawing process, focusing on optimizing process parameters to reduce maximum drawing force and wrinkling. MATLAB pattern search optimization and finite element simulations using ABAQUS/EXPLICIT were employed. Results showed that the optimized parameters effectively minimized drawing force and wrinkling compared to previous studies. Menghar et al^20^.. studied hydroforming deep drawing with radial pressure for AL-1050 sheets, demonstrating that optimized pressure paths improve thickness distribution but may increase thinning under excessive pressure. These approaches, while effective, introduce additional system complexity and are less suitable for conventional press-based manufacturing. Sekhara Reddy et al.^21^. investigated the deep drawing process of AA6111 aluminum alloy sheets to experimentally determine the Limiting Drawing Ratio (LDR) and validate the results with FE simulations. Using a lab-developed tool setup and Taguchi design of experiments, the maximum blank size without defects was identified, yielding an LDR of 1.8325. The FE simulations with Pam-Stamp showed good agreement with experiments, confirming that strains remained within the safe region for successful cups, while exceeding the limit for fractured or wrinkled ones.

Although extensive research has been conducted on deep drawing, many existing studies primarily address different forming techniques or non-aluminum materials and often examine process parameters individually rather than in combination. In particular, limited attention has been given to the combined influence of punch velocity, blank thickness, and blank holder force in the conventional cold deep drawing of low-depth aluminum cups.

Optimizing this process is industrially critical in automobile manufacturing, aerospace, electronic communications, and rail transit^22^. It is used to produce hollow structures such as faucets, bathtubs, and sinks, as well as cooking vessels, buckets, and cups. Thus, deep drawing has a wide range of applications across various industries^23^. In such applications, minimizing thinning, controlling peak forming force, maintaining stable strain distribution, and enhancing formability are essential to ensure dimensional accuracy, structural reliability, and reduced tooling iterations.

Despite these requirements, a predictive model capable of simultaneously evaluating these performance indicators under the coupled influence of key process parameters is still lacking.

## Methodology

The present study implemented an integrated numerical–statistical framework using a Taguchi design of experiments to systematically investigate the deep drawing of aluminum circular blanks, addressing the research gap. Numerical simulations were performed using LS-DYNA to evaluate the effects of punch velocity, blank thickness, and blank holder force on maximum thinning (%), maximum forming force, von Mises stress, effective plastic strain, and Forming Limit Diagram (FLD) plots. The simulation results were analyzed using ANOVA within the RSM framework to determine statistical significance and quantify interactions among the input parameters.

## Deep drawing theory analysis

Deep drawing is a sheet metal forming process in which a flat blank is transformed into a hollow component. The achievable drawing depth is limited by several factors, including material ductility, punch and die geometry, friction, and process parameters. Accurate evaluation of drawing force, holding force, and initial blank size is essential for successful forming^2^.

### Measures of drawing

One of the key indicators of the severity of a deep drawing process is the Drawing Ratio (D_R_). For a cylindrical cup, the drawing ratio is simply defined as the ratio of the blank diameter (D_b_) to the punch diameter (D_p_). Mathematically, it can be expressed as:

1 $$DR=\frac{{D}_{b}}{{D}_{p}}$$

### The drawing ratio

Offers a general, though approximate, measure of the severity of a deep drawing operation. A higher drawing ratio indicates a more severe forming condition. In practice, the maximum safe limit for the drawing ratio is around 2.0. However, the actual limiting value in each operation is strongly influenced by several factors, including the punch and die corner radii (R_p_ and R_d_), frictional conditions, the depth of draw, as well as the sheet metal’s characteristics such as ductility and anisotropy in its strength properties^2^. Another useful parameter for evaluating drawing operations is the reduction (r), defined as:

2 $$r=\frac{{{D}_{p}-D}_{b}}{{D}_{b}}$$

It is very closely related to drawing ratio. Consistent with the previous limit on DR (DR ≤ 2.0), the value of reduction r should be less than 0.50.

### A third important measure

In deep drawing is the thickness-to-diameter ratio (t/D_b_), which is defined as the thickness of the starting blank (t) divided by the blank diameter (D_b_). This ratio is often expressed as a percentage, and it is generally desirable for t/D_b_ to exceed 1%. When the ratio falls below this threshold, the likelihood of wrinkling during the forming process increases. If the limits associated with the drawing ratio, reduction, or thickness-to-diameter ratio are exceeded in the design of a drawn part, the forming operation must be carried out in two or more drawing stages, sometimes requiring intermediate annealing between stages to restore ductility and prevent failure^2^.

3 $$\frac{t}{{D}_{b}}\ge1\%$$

### Forces

The drawing force required for a given operation can be approximately estimated using the following expression:

4 $$F=\pi{D}_{p}t\left(TS\right)\left(\frac{{D}_{b}}{{D}_{p}}-0.7\right)$$

where F is the drawing force (N), t is the original blank thickness (mm or in), TS is the tensile strength of the material (MPa), and D_b_ and D_p_ represent the starting blank diameter and punch diameter, respectively (mm). The constant 0.7 serves as a correction factor to account for frictional effects during the process. This equation provides an estimate of the maximum force experienced during drawing. In practice, the drawing force is not constant but varies with the downward movement of the punch, typically reaching its peak value at approximately one-third of the punch stroke length^2^.

### The holding force

Plays a critical role in deep drawing operations, as it helps to control material flow and prevent wrinkling. As a rough approximation, the required holding pressure can be taken as about 0.015 of the yield strength of the sheet metal. This pressure is then multiplied by the area of the blank that is in contact with the blank holder to obtain the total holding force^2^. In equation form:

5 $${F}_{h}=0.015Y\pi\left\{{D}_{b}^{2}-{(D}_{p}+2.2t+2{R}_{d}{)}^{2}\right\}$$

where F_h_ = holding force in drawing, N; Y = yield strength of the sheet metal, MPa; t =starting stock thickness, mm (in); R_d_ = die corner radius, mm (in); and the other terms have been previously defined.

## Finite element analysis of deep drawing

The numerical simulations were performed using the LS-DYNA explicit solver, which has a long-established history in sheet metal forming simulations within the metal forming industry^24^. The explicit dynamic approach is particularly suitable for problems involving impact events or short-duration, high-pressure loading conditions. It is also well suited for highly complex and nonlinear analyses involving rapid material deformation or failure^25^, as encountered in the deep drawing process.

### Finite element model setup (geometry definition)

**Fig. 1:**
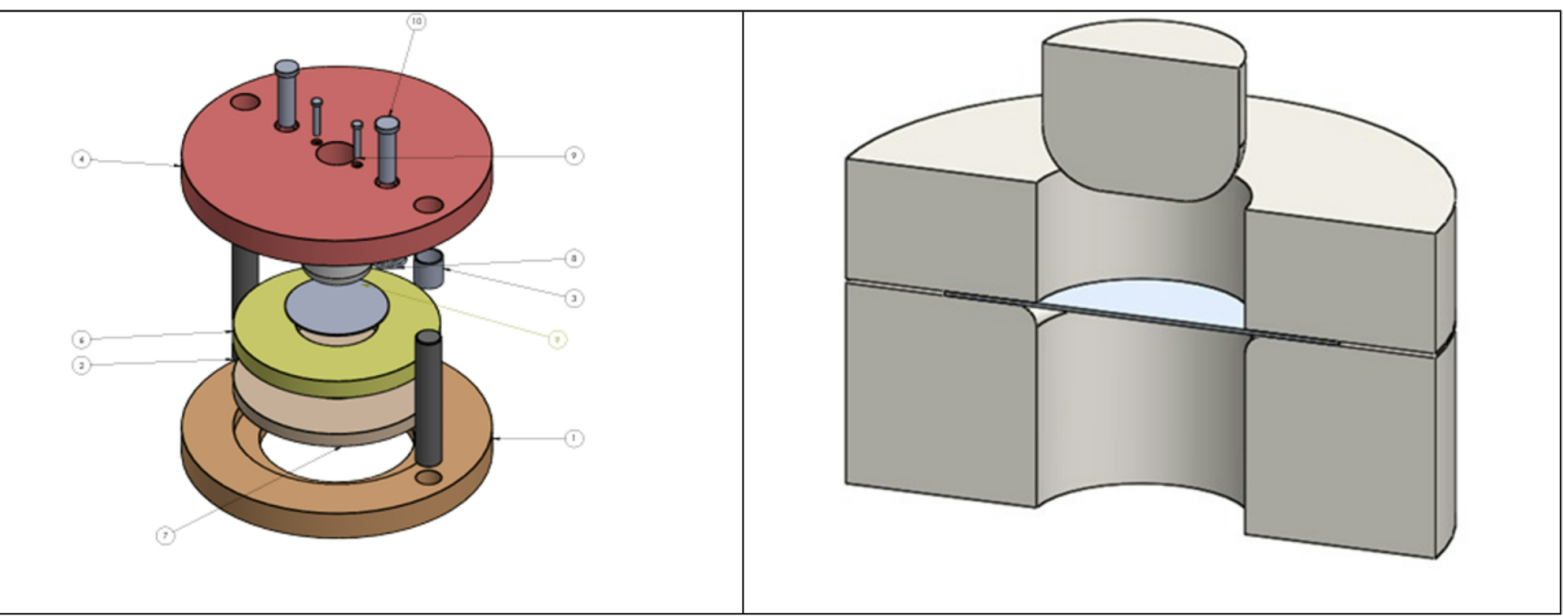
Deep Drawing Components.

The deep drawing setup consists of several key geometrical components designed to form aluminum sheets into desired shapes. The blank is an aluminum sheet with a diameter of 100 mm and varying thickness levels of 1 mm, 1.5 mm, and 2 mm. The punch, which applies the forming force, has a diameter of 50 mm and a fillet radius of 15 mm to ensure smooth material flow and reduce stress concentration. The die, which guides the material into shape, features an outer and inner diameter of 53 mm and a fillet radius of 5 mm to prevent tearing and wrinkling. Additionally, the blank holder, with an outer diameter of 150 mm, applies pressure to the sheet to control material flow and minimize defects during the drawing process. These components work together to achieve precise and efficient deep drawing operations as shown in Table 1; Fig. 1.

**Table 1:** Dimensions of deep drawing parts.

Parts	Values	Units
**Blank**	Aluminum sheet, 100 mm diameter, thickness levels: 1 mm, 1.5 mm, 2 mm.	mm
**Punch**	Diameter 50 mm, fillet radius 15 mm.	mm
**Die**	Outer and inner diameter 53 mm, fillet radius 5 mm.	mm
**Blank Holder**	Outer diameter 150 mm.	mm

### Material characteristics of Al‑1050

In this study, Al-1050 aluminum with a thickness of 1 mm,1.5 mm, and 2 mm were utilized as the blank material. The mechanical properties were characterized through tensile testing in accordance with the ASTM E8 standard^20^.

**Fig. 2:**
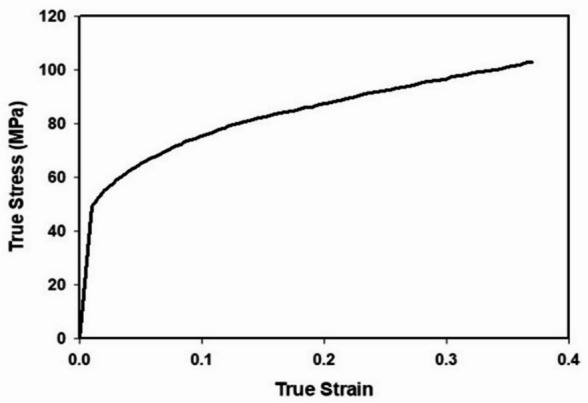
True stress and True strain obtained from experimental tensile test^20^.

The experimental tensile test results for Aluminum alloy Al-1050 are illustrated in Fig. 2, which presents the true stress–true strain relationship. The curve shows the material’s typical elastic–plastic behavior, starting with a steep linear region corresponding to elastic deformation, followed by yielding and subsequent strain hardening until failure^20^. The mechanical properties obtained from the test are summarized in Table 2. AL-1050 has a density of 2700 kg/m³, a Young’s modulus of 70 GPa, and a Poisson’s ratio of 0.33, indicating its relatively high stiffness and ductility. The material exhibits a yield stress of 38 MPa, beyond which plastic deformation occurs, and it follows a hardening coefficient of 0.24, reflecting its ability to sustain higher stresses with increasing strain. These results confirm that Al-1050 is a lightweight, ductile material with good formability, making it suitable for sheet metal forming applications.

**Table 2:** Mechanical properties of AL-1050^20^.

Density (ρ) (kg/m^3^)	2700
Young’s Modulus (E) [GPa]	**70**
Poisson ratio (*v*)	**0.33**
Hardening coefficient (n)	**0.24**
Yield stress (σ_y_) [MPa]	**38**

### Meshing

Shell elements were adopted instead of solid elements because, in the deep drawing process, the stress variation through the sheet thickness can be neglected, corresponding to a membrane stress state. This assumption significantly reduces computational time. However, shell elements do not capture the through-thickness deformation history^26^.

**Fig. 3:**
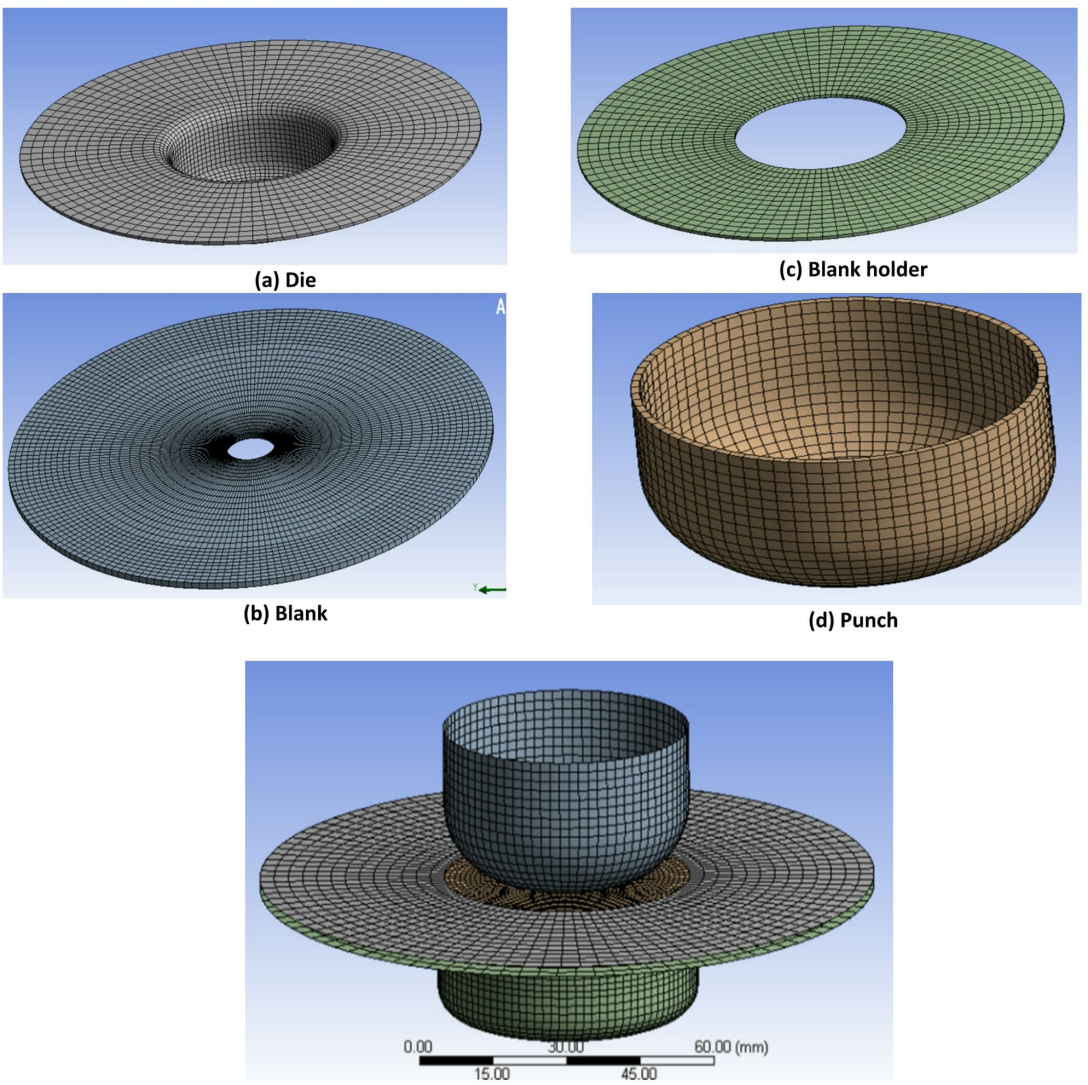
Meshing of deep drawing.

Figure 3 illustrates the finite element mesh generated for the deep drawing simulation using ANSYS. The model includes the punch (top), blank holder (middle ring), die (bottom), and the aluminum blank (gray circular sheet).

The deformable blank was modeled using Belytschko–Tsay quadrilateral shell elements with seven through-thickness integration points, while the rigid tools were modeled using the same formulation with three integration points^27^, the mesh properties are detailed in Table 3.

**Table 3:** Mesh properties of the FEM model.

No.	Part Name	Part Type	Element Order	Meshing Technique	Number of Elements	Number of Nodes
1	Die	Rigid	Linear	Edge sizing, mapped face meshing	4261	4311
2	Blank	Flexible	Linear	Edge sizing, mapped face meshing	2156	2233
3	Blank holder	Rigid	Linear	Edge sizing, mapped face meshing	1300	1400
4	Punch	Rigid	Linear	Edge sizing, mapped face meshing, uniform quad method	1760	1840

Aspect ratio in two-dimensional elements is defined as the ratio of the maximum edge length to the minimum edge length of an element (Aspect Ratio = maximum edge length/minimum edge length). The ideal value is 1.0, and values below 5 are generally considered acceptable^28^. In the present mesh, the aspect ratio ranged from 1.001 to 3.636, with an average value of 1.3427 and a standard deviation of 0.40248, indicating good element quality within the acceptable range.

### Boundary conditions in deep drawing simulation

In LS-DYNA, various material models are available, and two material cards were selected for this simulation. The die, punch, and blank holder were modeled as rigid bodies using MAT_RIGID (MAT_20). The deformable blank was modeled using the *MAT_PIECEWISE_LINEAR_PLASTICITY* (MAT_123) card, consistent with the previous study^29^.

**Fig. 4:**
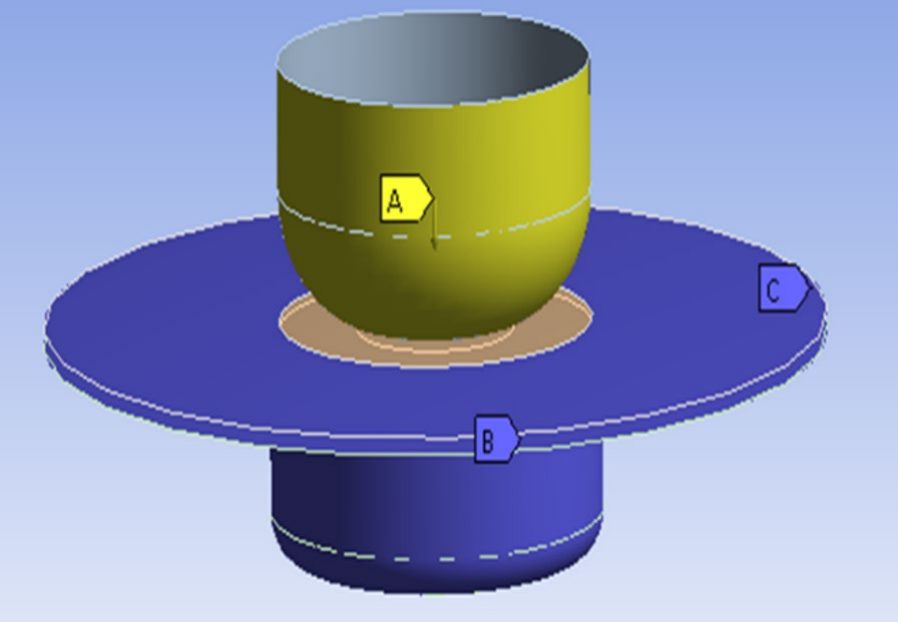
Boundary conditions of the deep drawing.

The boundary conditions applied in the deep drawing simulation are illustrated in Fig. 4. The punch (A) and blank holder (B) were constrained in the X and Y translations as well as in all rotational degrees of freedom (X, Y, Z), allowing only the prescribed downward motion along the Z-axis for both components. The die (C) was fully constrained in all translational and rotational degrees of freedom to ensure structural stability throughout the forming process.

The motion of the punch and the blank holder was defined in two steps. First, the velocity–time profile was specified using the *DEFINE_CURVE* card. Second, this curve was assigned to the punch through the *BOUNDARY_PRESCRIBED_MOTION_RIGID* card, with the appropriate axis and direction of motion defined^15^. Contact during forming was defined using the CONTACT_FORMING_ONE_WAY_SURFACE_TO_SURFACE_SMOOTH algorithm, where the rigid components were assigned as the master surfaces and the deformable blank was defined as the slave surface^30^.

### Taguchi design of experiments

The study investigated three critical process parameters in the deep drawing operation, each evaluated at three distinct levels. The parameter levels were selected based on ranges reported in previous studies punch velocity^31,32^–^33^, blank thickness^34^^35^,, and blank holder force^34^^35^, to ensure realistic and practically relevant conditions, as shown in Table 4.

**Table 4:** Deep drawing parameters and levels.

Parameters	unit	level 1	level 2	level 3
**Punch Velocity**	mm/s	2	5	8
**Thickness**	mm	1	1.5	2
**Blank Holder Force**	kN	5	10	15

To systematically analyze their influence on the process, the Taguchi method was employed for the design of experiments, resulting in an L9 orthogonal array covering the three parameters at three levels, which required only nine simulation runs. This approach minimized the number of experimental trials, thereby reducing the time needed, which in effect reduced the cost of finding the necessary information^36^.

## Results and discussions

### Finite element simulation results using LS-DYNA

This section presents the numerical results obtained from the finite element simulations and the design of experiments for the nine deep drawing cases. The influence of punch velocity, blank thickness, and blank holder force on key process indicators was systematically evaluated across all parameter combinations.

For each run, several output responses were recorded directly from LS-DYNA simulations, including maximum thinning (%), maximum forming force, Von Mises stress, and maximum plastic strain. Table 5 summarizes these FEM-generated outputs for each combination of process parameters. This dataset provides a solid basis for subsequent statistical analyses, such as ANOVA and response surface methodology (RSM), to determine the significance and interactions of the input parameters and identify optimal process conditions.

**Table 5:** FEM-generated results of the Taguchi L9 experiments.

No.	Process parameters			FEM-generated outputs			
	Punch Velocity (mm/s)	Thickness (mm)	Holder Force (KN)	Maximum Thinning (%)	Max Forming Force (KN)	Von Mises Stress (MPa)	Plastic Strain
1	2	1	5	31.55	8.19	137.37	0.706
2	2	1.5	10	33.83	12.4	145.48	0.774
3	2	2	15	38.96	31.2	149.9	0.821
4	5	1	10	23.07	7.67	116.25	0.482
5	5	1.5	15	25.21	11.5	123.22	0.552
6	5	2	5	36.86	28.0	157.07	0.89
7	8	1	15	19.46	6.16	107.06	0.404
8	8	1.5	5	20.26	10.5	112.16	0.441
9	8	2	10	20.83	16.7	112.76	0.447

#### Effective thickness distribution

**Fig. 5:**
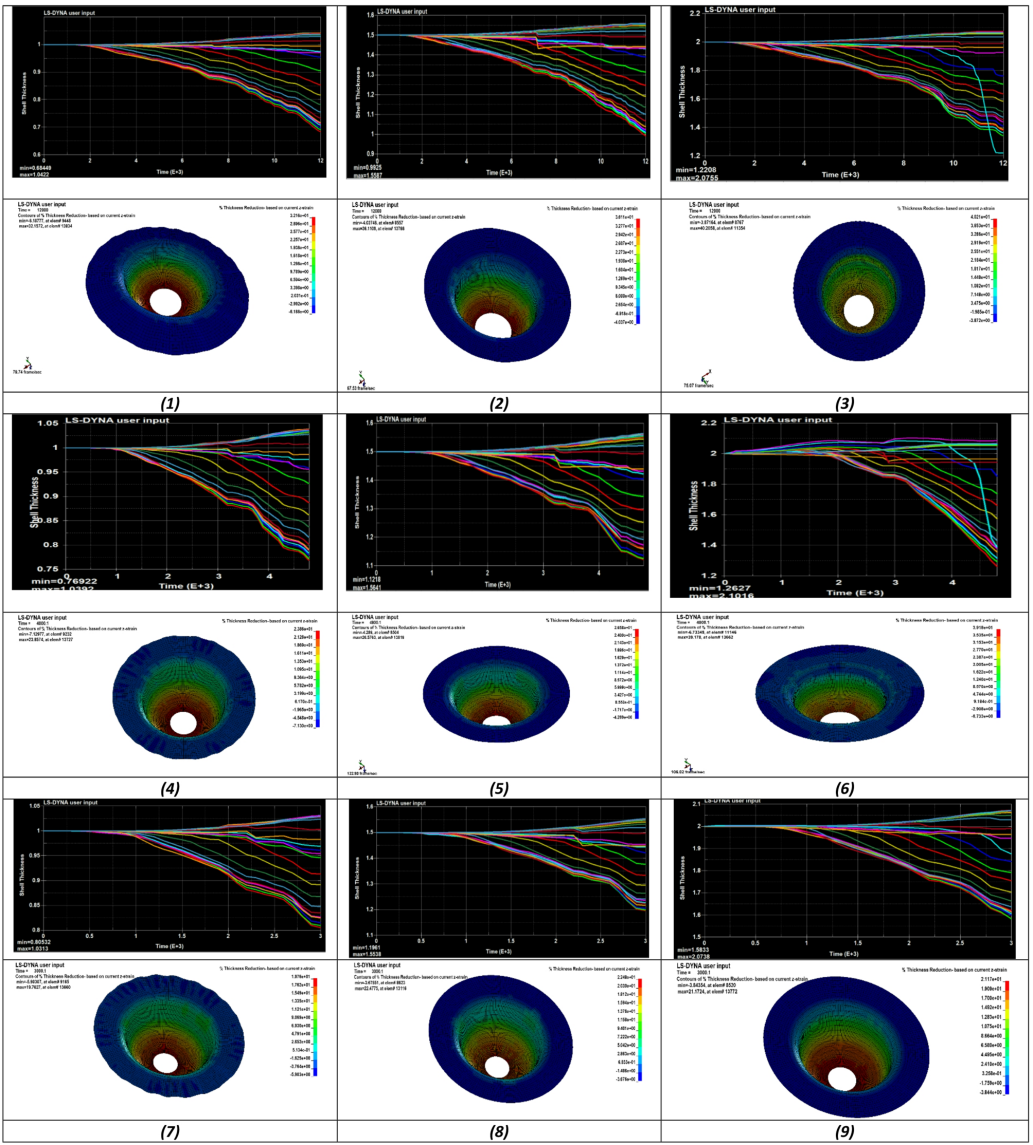
Result of thickness distribution on the sheet.

This section presents the thickness evolution during the deep drawing process. Due to the axisymmetric nature of the blank, an element path was defined from the center toward the outer edge to extract thickness data. Figure 5 illustrates the variation of shell thickness with simulation time obtained from LS-DYNA for all experiments. The horizontal axis represents time, while the vertical axis denotes shell thickness. Each colored curve corresponds to an element located along the defined path, showing its thickness variation throughout the forming process.

The results indicate a progressive reduction in thickness with increasing time. Across all experiments, the maximum thinning occurs in the region adjacent to the punch nose radius, representing the most critical deformation zone. This area follows the punch profile and experiences the highest strain concentration. The pronounced thickness reduction is primarily governed by radial tensile deformation as the material flows over the punch radius. In contrast, the flange region exhibits relatively limited thinning due to its proximity to the undeformed zone, which promotes thickness preservation.

The results show a clear variation in maximum thinning across the three groups. For runs 1–3 (punch velocity = 2 mm/s), thinning ranges from 31.55% to 38.96%, representing the highest values. For runs 4–6 (5 mm/s), thinning decreases to a range of 23.08% to 36.87%. In runs 7–9 (8 mm/s), the thinning values are the lowest and more stable, ranging from 19.47% to 20.84%.

Punch velocity shows an inverse relationship with thinning, as increasing the velocity from 2 mm/s to 8 mm/s reduces the thinning range from 31.55 to 38.96% to 19.47–20.84%^34^. Blank thickness exhibits a direct relationship with thinning, where increasing thickness from 1 mm to 2 mm consistently increases the thinning percentage within each velocity group^37^. Blank holder force also influences thinning, with higher holding forces generally contributing to increased thinning at lower velocities^38^.

The simulation results not only identify critical zones prone to excessive thinning but also reveal the sensitivity of wall thickness to process parameters, providing guidance for die design and parameter optimization. By understanding the interplay of punch velocity, blank thickness, and blank holder force, designers can minimize tearing risk and achieve more uniform wall thickness in deep drawing operations.

#### Effective maximum forming force distribution

**Fig. 6:**
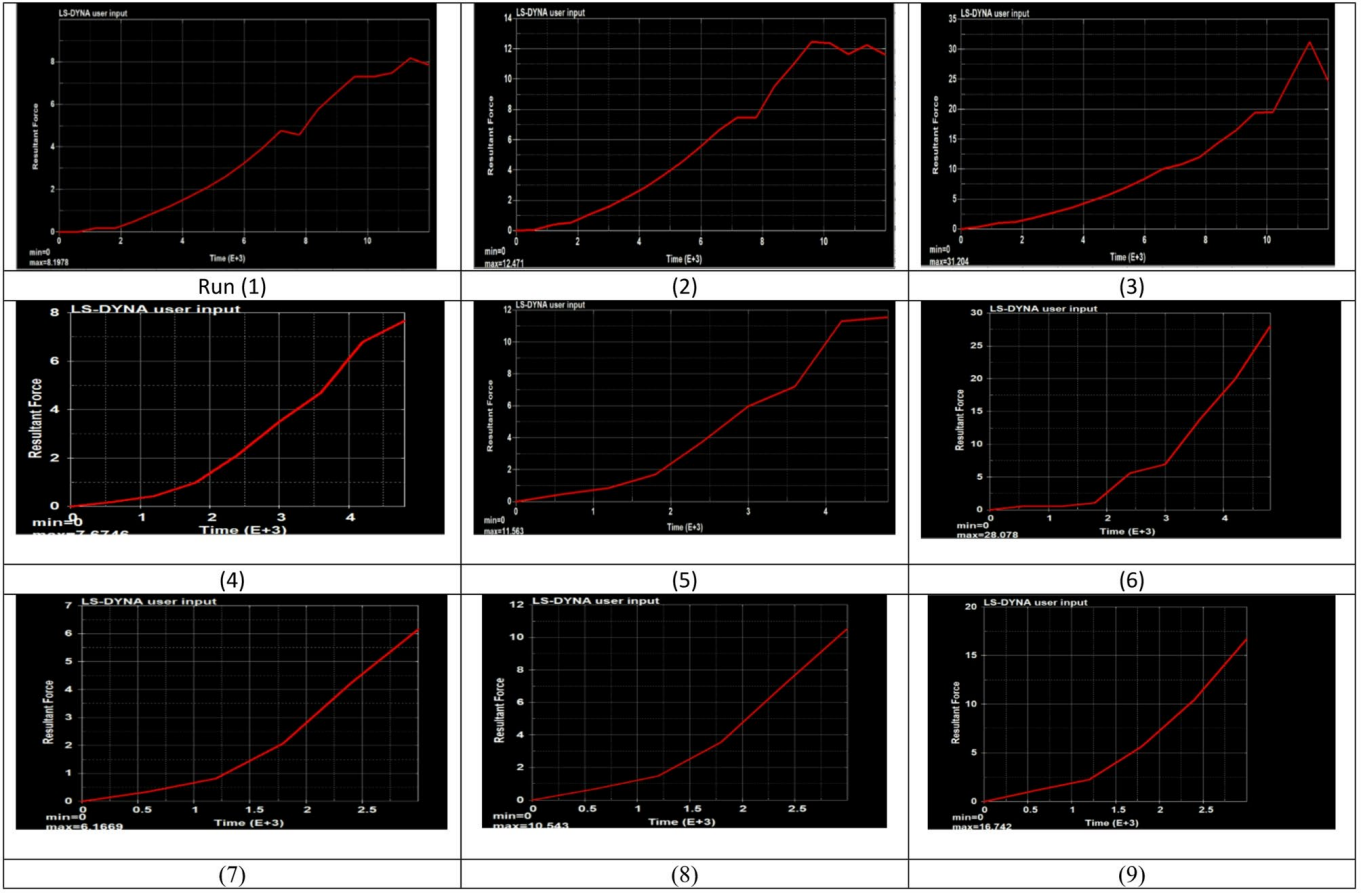
Result of force distribution on the sheet.

The forming force represents the resistance of the sheet material to plastic deformation during the deep drawing process. As shown in Fig. 6, the forming force increases with punch displacement. This increase is primarily governed by the material’s strain-hardening behavior^39^, as plastic deformation progresses during drawing. As the punch advances, the material undergoes work hardening, leading to an increase in flow stress. Consequently, a higher force is required to continue deformation, compensating for the reduction in cross-sectional area and ensuring stable material flow throughout the process.

The results show a clear variation in maximum forming force across the three velocity groups. For runs 1–3 (punch velocity = 2 mm/s), the forming force ranges from 8.19 kN to 31.2 kN, representing the highest variation among all groups. In runs 4–6 (5 mm/s), the forming force decreases to a range of 7.67 kN to 28.0 kN. For runs 7–9 (8 mm/s), the forming force values are the lowest and more stable, ranging from 6.16 kN to 16.7 kN.

Punch velocity exhibits an inverse relationship with maximum forming force, as increasing the velocity from 2 mm/s to 8 mm/s generally reduces the forming force^40^. In contrast, blank thickness shows a direct correlation with forming force, with thicker sheets requiring higher forces due to the increased material volume resisting deformation^34^. Blank holder force also influences the forming force: higher holding forces increase friction in the flange region, raising the tensile stress in the wall area and consequently increasing the required forming force^8^.

The ideal forming force is defined as the sufficient force required to plastically deform the blank without inducing excessive thinning or fracture, thereby ensuring improved formability. Based on the results presented in Table 5, this condition corresponds to Experiment 7, conducted at a punch velocity of 8 mm/s, a blank thickness of 1 mm, and a blank holder force of 15 KN. Under these conditions, a lower thinning percentage of 19.468% was obtained, with a corresponding forming force of 6.16 KN, indicating a stable and safe deformation state capable of achieving the desired shape.

#### Effective plastic strain distribution

**Fig. 7:**
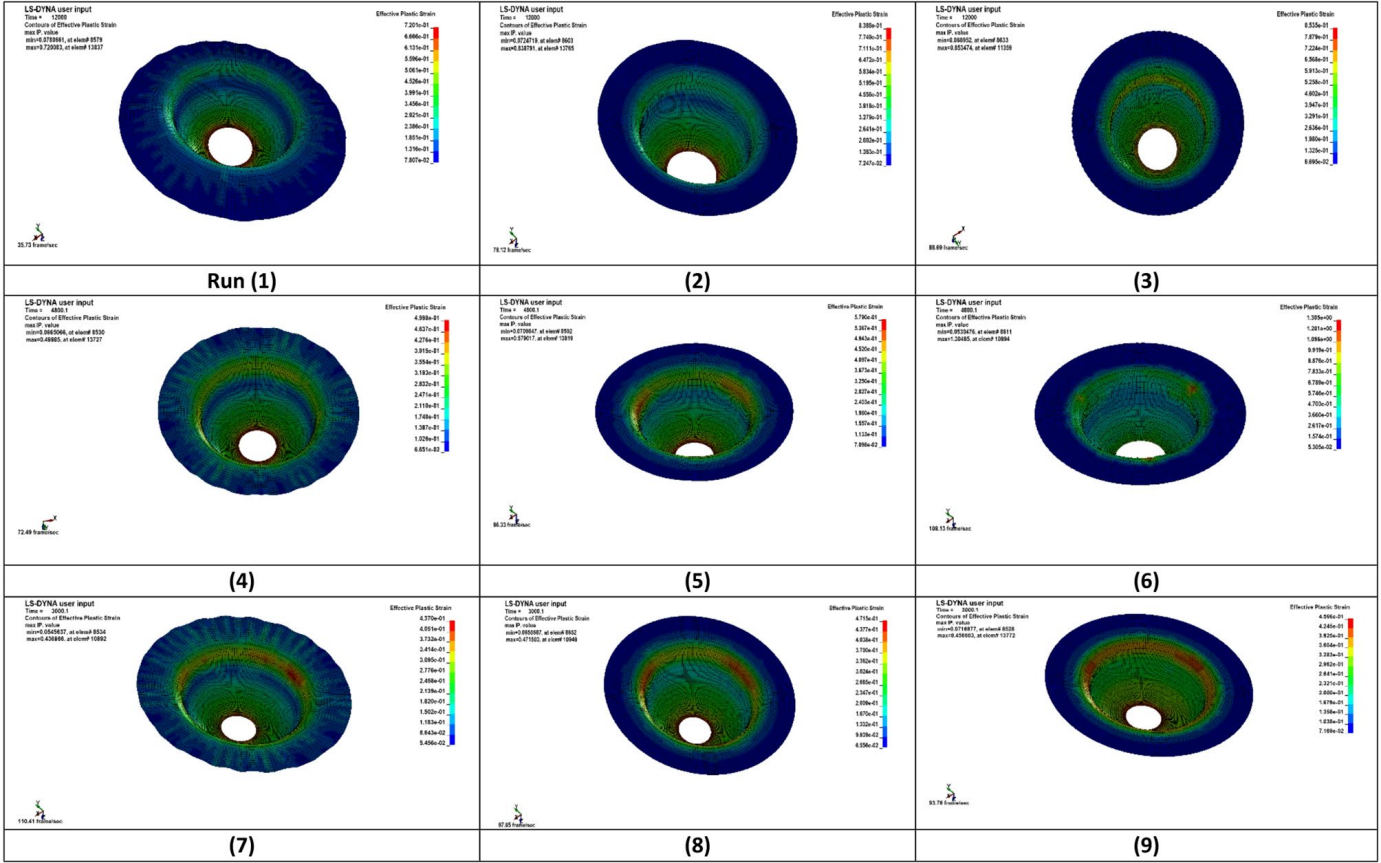
Result of plastic strain distribution on the sheet.

Figure 7 displays the contour plot of effective plastic strain at the final stage of the deep drawing simulation using LS-DYNA. The contour map illustrates the distribution of accumulated plastic deformation across the deformed blank. The results show that the plastic strain is highly concentrated around the die radius and punch contact area, which is consistent with expected deformation behavior in deep drawing processes. Lower strain values are observed near the flange and outer regions, where less material flow and deformation occur. This analysis provides valuable insights into material flow characteristics and helps identify zones of high plastic work that may require process optimization or material strengthening.

The results indicate a clear variation in maximum plastic strain across the three punch velocity groups. For runs 1–3 (punch velocity = 2 mm/s), the plastic strain ranges from 0.706 to 0.821, representing relatively high deformation levels. In runs 4–6 (5 mm/s), the plastic strain values vary more widely, from 0.482 to 0.890, where run 6 exhibits the highest plastic strain among all experiments. For runs 7–9 (8 mm/s), the plastic strain values are the lowest and more consistent, ranging from 0.404 to 0.447, indicating improved deformation stability at higher velocities.

Punch velocity shows an overall inverse relationship with plastic strain, as increasing the velocity from 2 mm/s to 8 mm/s generally reduces the accumulated plastic deformation. Blank thickness exhibits a direct influence on plastic strain; thicker sheets (2 mm) consistently result in higher plastic strain values due to increased resistance to deformation and elevated stress levels. Blank holder force (BHF) also affects the plastic strain distribution. Higher holding forces tend to increase tensile stresses in the wall region through elevated frictional restraint in the flange, which in some cases promotes localized plastic deformation.

## Effective FLD analysis

Formability is defined as the maximum extent of plastic deformation that a material can undergo during a forming process without the occurrence of failure or instability mechanisms, such as cracking, localized necking, buckling, or fold formation^41^. It is commonly evaluated using forming limit diagrams (FLDs), which are obtained from standardized tests conducted under different strain paths until localized necking occurs. These diagrams enable quantitative comparison between sheet materials by identifying the strain combinations that lead to instability. In constructing the forming limit curve (FLC), deformation is experimentally measured using a circular grid applied to the sheet surface prior to forming, typically by printing or etching. As deformation proceeds, the circles transform into ellipses, reflecting the imposed strain state^42^.

The diameters of the deformed ellipses were measured near the fracture region and subsequently converted into true strain values to establish the forming limit curve (FLC). The principal major and minor strains were calculated from the measured ellipse diameters (D and d) using Eqs. (6) and (7)^43^.

6 $${\epsilon}_{1}=\mathrm{ln}\left(\frac{D}{{d}_{0}}\right)$$

7 $${\epsilon}_{2}=\mathrm{ln}\left(\frac{d}{{d}_{0}}\right)$$

where ${d}_{0}$ is the diameter of the circle before to the experiment.

**Fig. 8:**
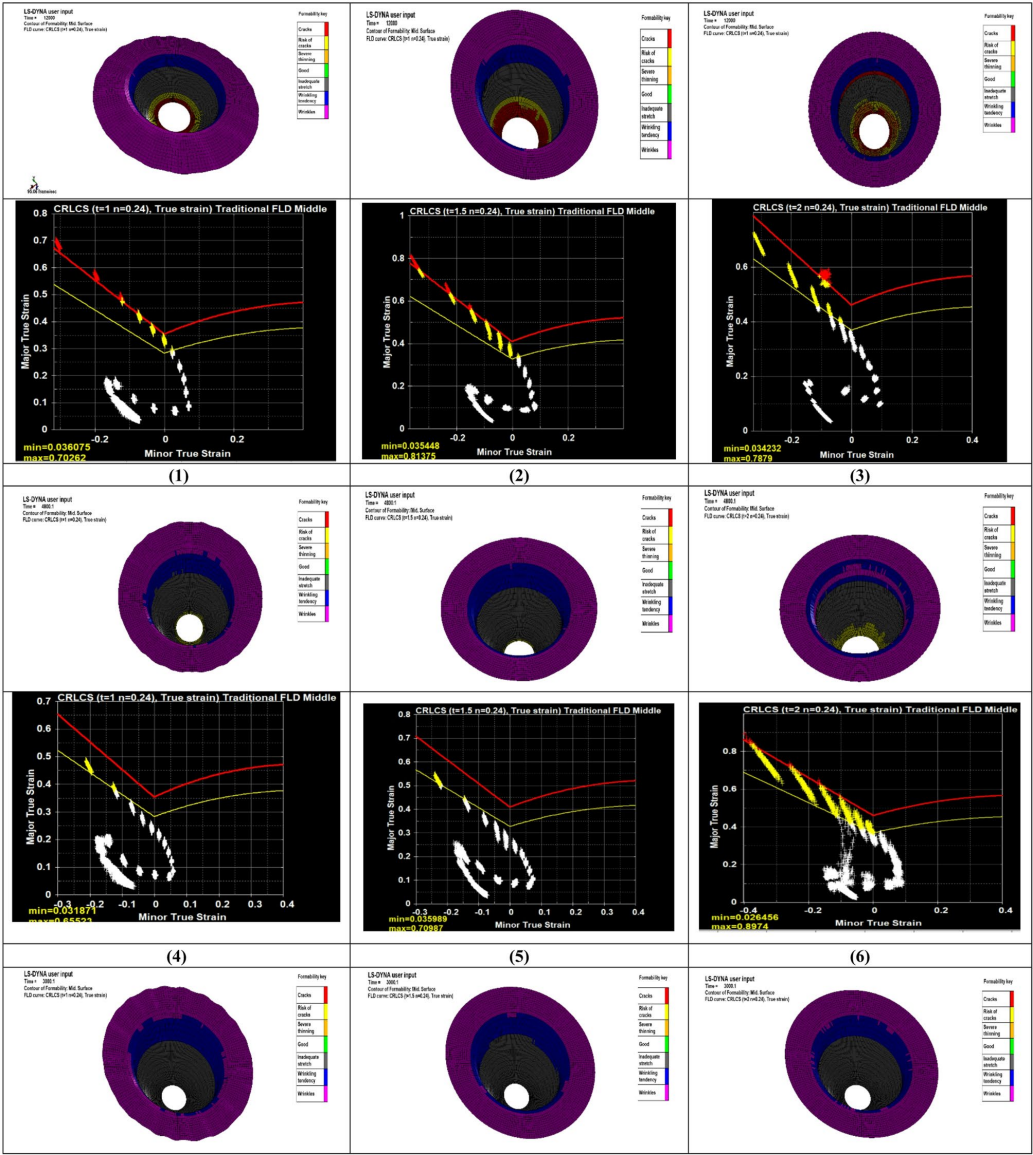

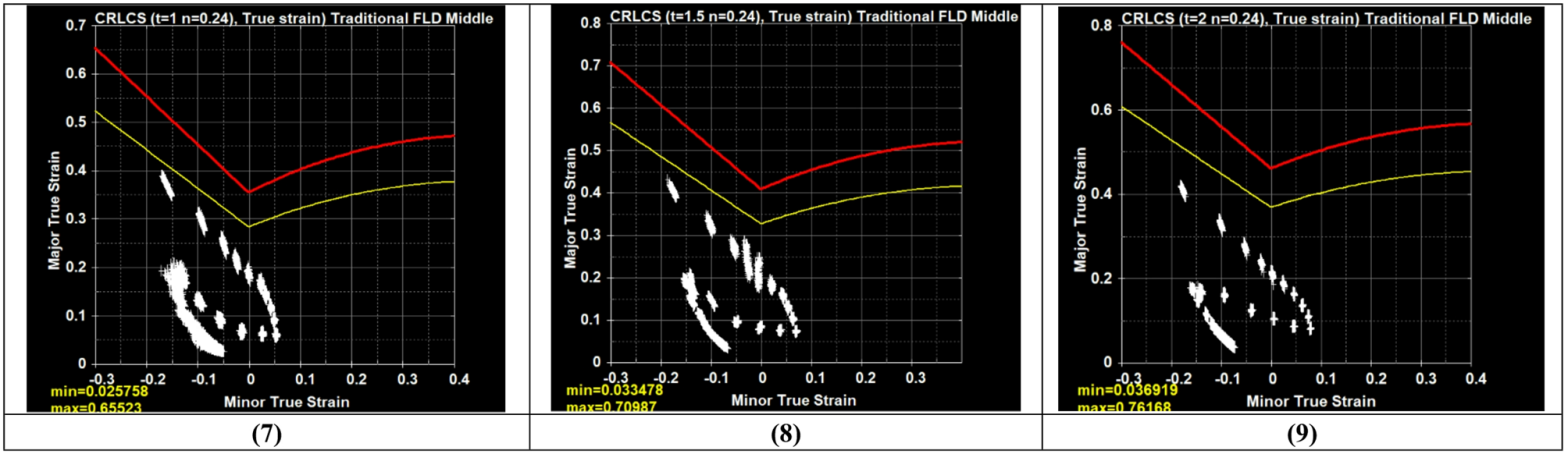
Result of FLD distribution on the sheet.

The results related to the FLD will be presented and analyzed in this section. Figure 8 shows the Forming Limit Diagram (FLD) generated using LS-DYNA. The diagram plots the major versus minor true strains of the blank shell elements during the deep drawing simulation. Both the safety margin curve and the forming limit curve are defined based on the sheet thickness (t) and material properties, particularly the strain-hardening exponent (n).

Within the diagram, the forming limit curve (FLC), illustrated in red, defines the critical boundary separating stable deformation from failure. The safety margin curve, shown in yellow, represents a conservative threshold below the FLC, providing an additional safety criterion for evaluating formability.

The forming limit diagrams for all experimental conditions exhibited consistent strain distributions. Most strain points were located in the negative minor strain region with moderate major strain values, indicating a drawing-dominated deformation mode close to uniaxial tension. A limited number of strain points appeared in the positive minor strain region, suggesting that biaxial stretching was not the dominant deformation mechanism in the present deep drawing process. Additionally, several points were observed toward the uniaxial compression region, reflecting localized compressive behavior in the flange area due to material flow during drawing. The observed scattered strain band reflects the non-uniform strain paths along the cup wall and flange. This behavior is consistent with the mechanics of deep drawing, as reported in^44,45^.

For runs 1–3 (punch velocity = 2 mm/s), several strain points approach the FLC, indicating the highest forming severity. In runs 4–6 (5 mm/s), the strain distribution shifts moderately away from the FLC, reflecting improved deformation stability. Runs 7–9 (8 mm/s) exhibit the safest behavior, with strain points well below the FLC and a more compact, stable distribution. Punch velocity exhibits an inverse relationship with forming severity, as increasing velocity promotes smoother material flow, reduces localized thinning, and delays approach to the forming limit. Blank thickness shows a direct effect, where thicker sheets (2 mm) lead to strain states closer to the FLC, while thinner sheets (1 mm) maintain larger safety margins. Blank holder force (BHF) has a secondary influence; lower forces (5 kN) allow more widely spread strain paths, moderate forces (10 kN) stabilize the distribution, and higher forces (15 kN) slightly shift strain points toward the FLC, likely due to increased friction and tensile stress.

The FLC serves as a key reference in this analysis, defining the limit of safe deformation. By relating strain states to the FLC, the results clearly indicate how process parameters influence forming severity and localized thinning, enabling identification of critical conditions and guiding optimization of the deep drawing process.

### Regression model

The regression model for key formability indicators in the deep drawing process such as maximum thinning percentage, maximum forming force, and plastic strain was developed based on the numerical results obtained from finite element analysis (FEA) simulations, as presented in Table 5. Statistical analysis and experimental design were performed using Design-Expert^®^ software (Version 13)^46^.

Table 6 presents the ANOVA results for the linear model developed to evaluate the maximum thinning percentage. The model is statistically significant, as evidenced by an F-value of 12.99 and a p-value of 0.0085, confirming the adequacy of the selected factors in predicting thinning behavior. Punch velocity (A) is identified as the most influential parameter, exhibiting a highly significant effect with an F-value of 30.47 and a p-value of 0.0027. Blank thickness (B) also shows a statistically significant contribution (*p* = 0.0360), although its influence is less dominant compared to punch velocity. In contrast, holder force (C) has an insignificant effect on maximum thinning (*p* = 0.5535). The relatively low residual error indicates good agreement between the model predictions and the numerical results within the investigated range.

**Table 6:** ANOVA for Linear model Maximum Thinning (%).

Source	Sum of Squares	df	Mean Square	F-value	*p*-value	
**Model**	408.54	3	136.18	12.99	0.0085	significant
A-Punch Velocity	319.47	1	319.47	30.47	0.0027	
B-Blank Thickness	84.85	1	84.85	8.09	0.0360	
C-Holder Force	4.22	1	4.22	0.4029	0.5535	
**Residual**	52.42	5	10.48			
**Cor Total**	460.96	8				

Table 7 presents the analysis of variance (ANOVA) for the linear model developed to predict the maximum forming force. The model is statistically significant with an F-value of 8.22 and a p-value of 0.0223, indicating that the selected process parameters have a meaningful influence on the response. Among the investigated factors, blank thickness (B) is identified as the most influential parameter, exhibiting a highly significant effect on the maximum forming force with an F-value of 22.03 and a p-value of 0.0054. In contrast, punch velocity (A) shows a limited and statistically insignificant contribution (*p* = 0.1693), while holder force (C) has a negligible effect on the response (*p* = 0.8575). The residual error is relatively low, confirming an acceptable fit of the linear model within the investigated design space. These results suggest that variations in maximum forming force are predominantly governed by blank thickness, whereas punch velocity and holder force play secondary roles under the studied conditions.

**Table 7:** ANOVA for Linear model Max forming Force.

Source	Sum of Squares	df	Mean Square	F-value	*p*-value	
**Model**	541.24	3	180.41	8.22	0.0223	significant
A-Punch Velocity	56.61	1	56.61	2.58	0.1693	
B-Blank Thickness	483.84	1	483.84	22.03	0.0054	
C-Holder Force	0.7848	1	0.7848	0.0357	0.8575	
**Residual**	109.80	5	21.96			
**Cor Total**	651.04	8				

Table 8 summarizes the analysis of variance (ANOVA) for the linear model developed to evaluate the von Mises stress. The obtained model is statistically significant, as indicated by an F-value of 9.93 and a p-value of 0.0151, confirming the adequacy of the selected factors in describing the stress response. Punch velocity (A) is identified as the dominant parameter influencing the von Mises stress, showing a highly significant effect with an F-value of 21.10 and a p-value of 0.0059. Blank thickness (B) also exhibits a statistically significant contribution (*p* = 0.0432), although its influence is less pronounced compared to punch velocity. Conversely, holder force (C) demonstrates a statistically insignificant effect on the von Mises stress (*p* = 0.2824). The relatively low residual error indicates a satisfactory agreement between the model predictions and the numerical results within the investigated parameter range. These findings highlight that stress evolution during the forming process is primarily governed by punch velocity and blank thickness, while the influence of holder force remains limited under the studied conditions.

**Table 8:** ANOVA for Linear model Von Mises Stress.

Source	Sum of Squares	df	Mean Square	F-value	*p*-value	
**Model**	0.0024	3	0.0008	9.93	0.0151	significant
A-Punch Velocity	0.0017	1	0.0017	21.10	0.0059	
B-Blank Thickness	0.0006	1	0.0006	7.24	0.0432	
C-Holder Force	0.0001	1	0.0001	1.45	0.2824	
**Residual**	0.0004	5	0.0001			
**Cor Total**	0.0028	8				

Table 9 presents the analysis of variance (ANOVA) for the linear model developed to predict the equivalent plastic strain. The proposed model is statistically significant, with an F-value of 9.32 and a p-value of 0.0173, indicating that the selected process parameters adequately describe the plastic deformation behavior. Punch velocity (A) is identified as the most influential factor, exhibiting a highly significant effect on plastic strain with an F-value of 20.24 and a p-value of 0.0064. Blank thickness (B) shows a marginal influence, being close to the significance threshold (*p* = 0.0530), suggesting a moderate contribution to plastic strain evolution. In contrast, holder force (C) has a statistically insignificant effect (*p* = 0.2987). The relatively low residual error confirms the adequacy of the linear model within the investigated parameter space. These results indicate that plastic strain development during the forming process is primarily governed by punch velocity, while the effects of blank thickness and holder force remain secondary under the studied conditions.

**Table 9:** ANOVA for Linear model Plastic Strain.

Source	Sum of Squares	df	Mean Square	F-value	*p*-value	
**Model**	0.2343	3	0.0781	9.32	0.0173	significant
A-Punch Velocity	0.1697	1	0.1697	20.24	0.0064	
B-Blank Thickness	0.0534	1	0.0534	6.37	0.0530	
C-Holder Force	0.0113	1	0.0113	1.34	0.2987	
**Residual**	0.0419	5	0.0084			
**Cor Total**	0.2763	8				

Table 10 presents the statistical indicators used to evaluate the adequacy and predictive performance of the developed linear models for Maximum Thinning (%), maximum forming force, von Mises stress, and plastic strain. The obtained coefficients of determination (R²) are relatively high, reaching 0.8863 for maximum thinning (%) and ranging between 0.8313 and 0.8563 for the remaining responses, indicating a strong correlation between the predicted and actual values. The adjusted R² values remain in reasonable agreement with R², confirming the statistical reliability of the models. Although the predicted R² values are comparatively lower, particularly for maximum forming force, von Mises stress, and plastic strain, they still demonstrate acceptable predictive capability within the investigated design space. The Adequate Precision values exceed the recommended threshold of 4 for all responses, confirming an adequate signal-to-noise ratio and the suitability of the models for navigation within the design domain. Furthermore, the relatively low coefficients of variation (C.V.%) and standard deviation values indicate good experimental consistency and limited data scatter.

**Table 10:** Model Adequacy and Predictive Performance Statistics.

Std. Dev.	Maximum Thinning (%)	Max forming Force	Von Mises Stress	Plastic Strain		Maximum Thinning (%)	Max forming Force	Von Mises Stress	Plastic Strain
	3.24	4.69	0.0090	0.0916	*R*²	0.8863	0.8313	0.8563	0.8482
**Mean**	27.78	14.70	0.1290	0.6130	**Adjusted R²**	0.8180	0.7301	0.7701	0.7572
**C.V. %**	11.65	31.87	6.94	14.94	**Predicted R²**	0.5440	0.4077	0.4268	0.3934
					**Adeq Precision**	10.2448	7.7151	8.9218	8.5999

**Fig. 9:**
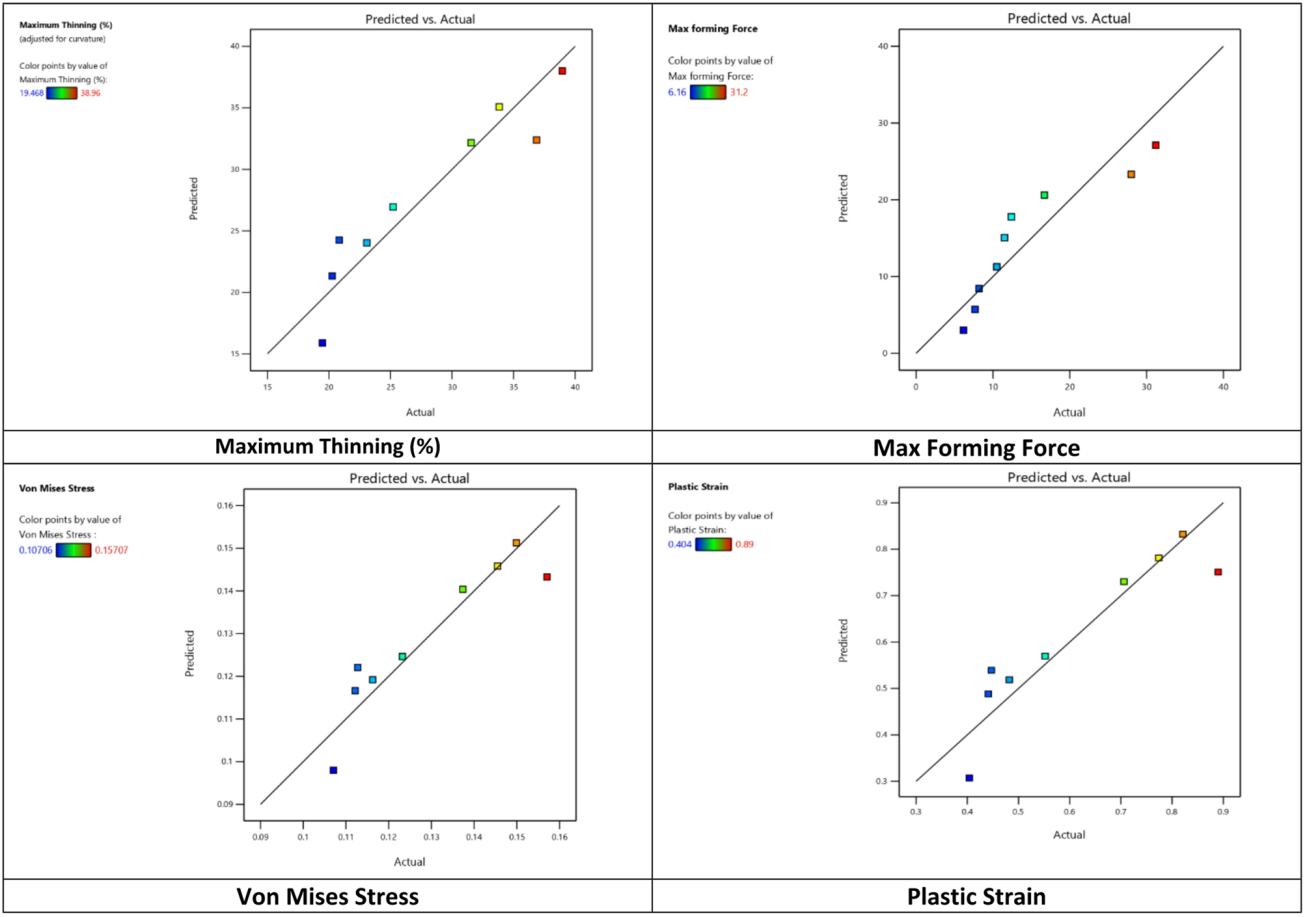
Predicted versus actual values for response in the deep drawing process.

The Predicted vs. Actual plot demonstrates a strong correlation between the model’s predicted values and the actual experimental data as shown in Fig. 9. Most of the data points lie very close to the diagonal line, indicating that the developed model provides highly accurate predictions. The color gradient, ranging from lower values of thickness to higher values of R1, further highlights the distribution of the results across the studied range. Only one point at the higher end shows a slight deviation from the line, which may be considered as a potential outlier.

#### Effect of process parameters on maximum thinning% distribution in deep drawing

**Fig. 10:**
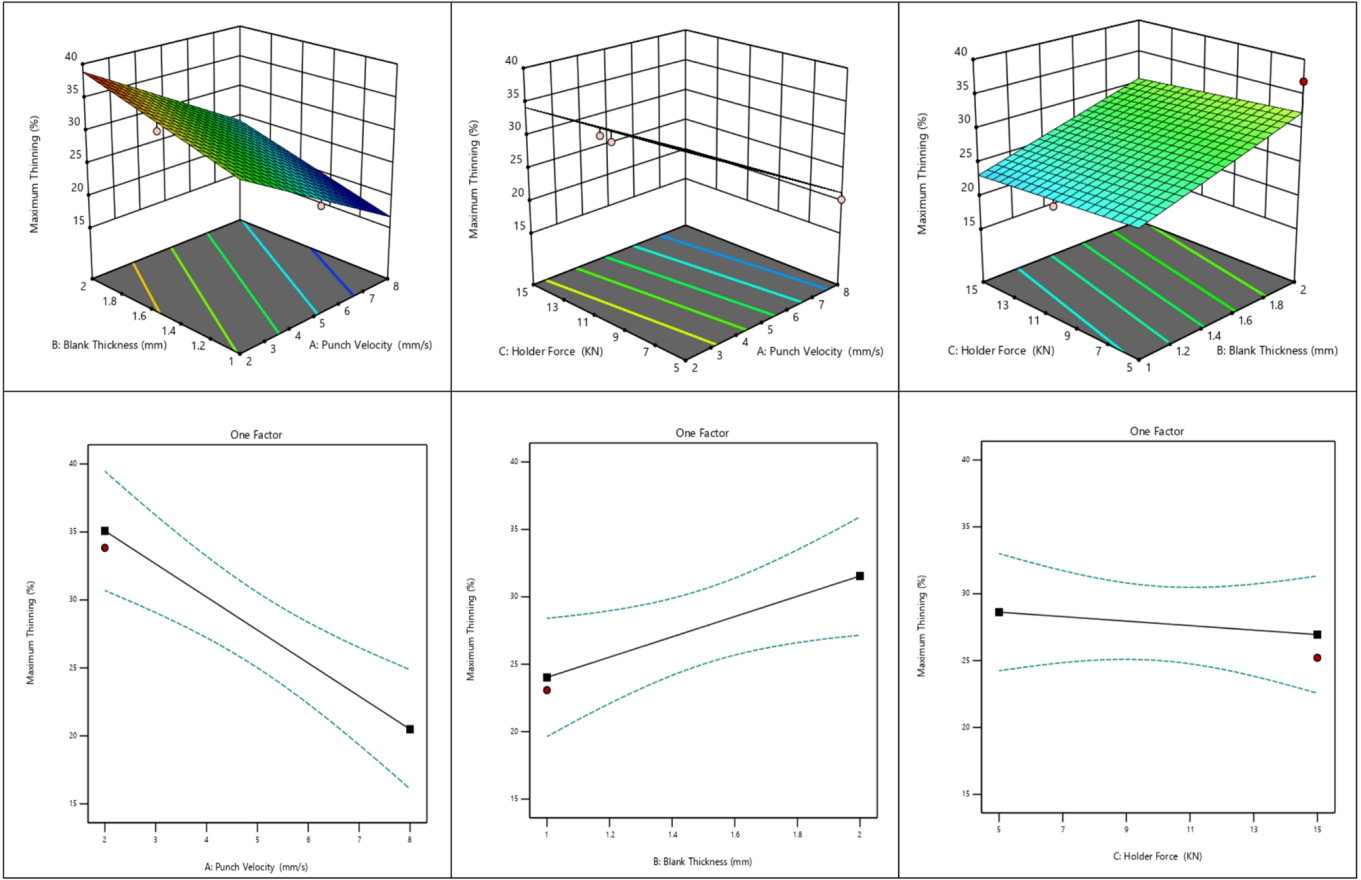
Effect of process parameters on maximum thininng % distribution in deep drawing.

Figure 10 shows the influence of punch velocity, blank thickness, and blank holder force on the maximum thinning percentage during deep drawing. The 3D surfaces indicate that higher punch velocity significantly reduces thinning. In contrast, increasing blank thickness increases thinning. The blank holder force has a moderate effect, slightly reducing thinning. The one-factor plots confirm these trends: thinning decreases markedly with punch velocity, increases with blank thickness, and slightly declines with higher blank holder force.

The variation in maximum thinning is consistently reflected in the numerical results, response plots, and statistical analysis. The thinning values decrease progressively with increasing punch velocity, indicating its dominant influence. Maximum thinning increases with blank thickness, whereas blank holder force shows a slight inverse relationship with thinning.

This behavior can be attributed to the reduced strain localization at higher punch velocities, which promotes more uniform material flow. The increase in thinning with blank thickness is associated with higher deformation resistance, leading to greater tensile stresses in the cup wall. In contrast, a higher blank holder force suppresses excessive flange flow and stabilizes material deformation, thereby slightly reducing thinning.

#### Effect of process parameters on max forming force distribution in deep drawing

**Fig. 11:**
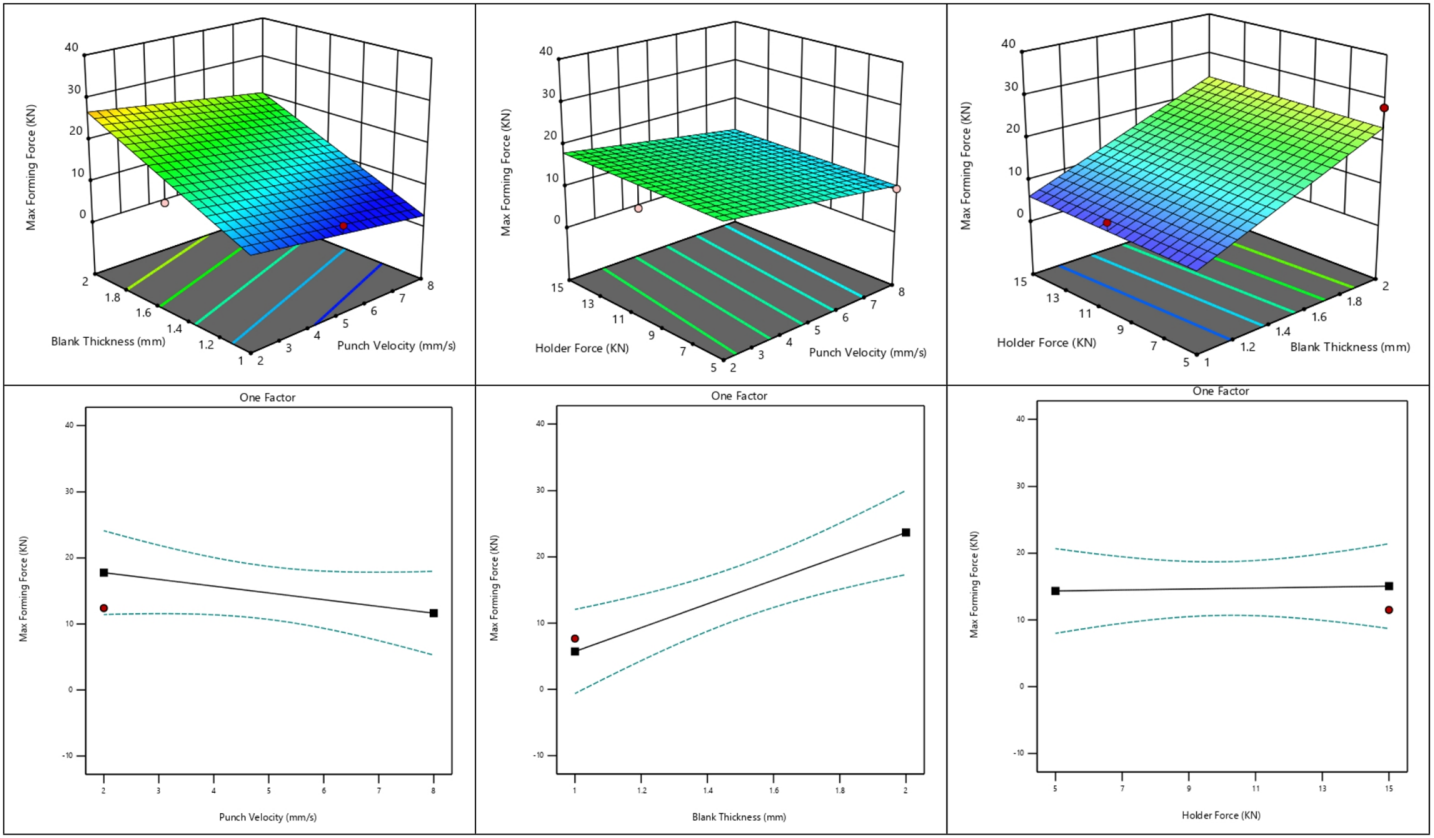
Effect of process parameters on max forming force in deep drawing.

Figure 11 illustrate the influence of the main process parameters punch velocity, blank thickness, and holder force on the maximum forming force during the deep drawing process. The 3D surface plots reveal that increasing blank thickness significantly raises the forming force due to the higher resistance of the material to deformation, while increasing punch velocity tends to reduce the required force. In contrast, the effect of holder force appears relatively small compared to the other parameters. The corresponding one-factor plots confirm these observations: maximum forming force increases clearly with greater blank thickness, decreases with higher punch velocity, and remains almost constant with variations in holder force. These results indicate that blank thickness is the dominant factor influencing forming force, followed by punch velocity, whereas holder force plays only a minor role.

The variation in maximum forming force is clearly supported by the numerical results, response surface plots, and statistical analysis. The forming force increases markedly with increasing blank thickness, confirming its dominant effect on the process. In contrast, the maximum force decreases with higher punch velocity, while blank holder force exhibits an almost negligible influence. These trends indicate that blank thickness governs the deformation load, followed by punch velocity, whereas holder force plays a minor role.

This behavior is mainly attributed to the higher deformation resistance associated with thicker blanks, which requires greater forming load. The reduction in force at higher punch velocities may be related to improved material flow and reduced frictional sticking during deformation. Meanwhile, the limited sensitivity to blank holder force suggests that flange constraint was already sufficient within the tested range, resulting in only a marginal effect on the overall forming load.

#### Effect of process parameters on von mises stress distribution in deep drawing

**Fig. 12:**
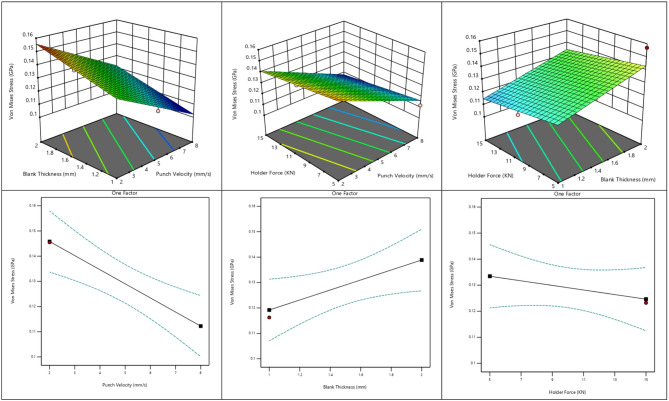
Effect of process parameters on Von Mises stress distribution in deep drawing.

The response surface and one-factor plots illustrate the effect of punch velocity, blank thickness, and holder force on the Von Mises stress distribution in the deep drawing process as shown Fig. 12. The results indicate that increasing punch velocity leads to a significant reduction in Von Mises stress, suggesting that higher drawing speeds facilitate material flow and reduce resistance to deformation. Conversely, increasing blank thickness results in a clear rise in stress levels due to the higher material strength and resistance to plastic deformation. The influence of holder force is minor, with only slight variations in stress observed across the studied range. Overall, blank thickness is identified as the most dominant factor influencing the stress distribution, followed by punch velocity, while holder force has a limited effect.

The variation in von Mises stress is consistently supported by the numerical results, response plots, and statistical analysis. The stress level decreases noticeably with increasing punch velocity, indicating its strong influence on the deformation behavior. Conversely, von Mises stress increases with increasing blank thickness, while blank holder force exhibits only a marginal reducing effect across the investigated range. These observations suggest that punch velocity and blank thickness are the primary factors governing the stress state, whereas holder force plays a limited role.

This behavior can be explained by the reduced strain concentration at higher punch velocities, which promotes smoother material flow and lowers the effective stress. The increase in stress with blank thickness is attributed to the higher resistance to plastic deformation offered by thicker sheets. In contrast, the minimal effect of blank holder force indicates that flange restraint was sufficient within the studied conditions, resulting in only slight stress relief.

#### Effect of process parameters on plastic strain distribution in deep drawing

**Fig. 13:**
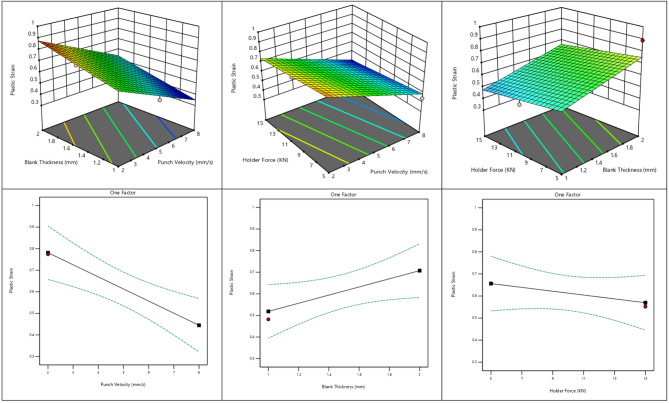
Effect of process parameters on plastic strain distribution in deep drawing.

The response surface and one-factor plots present the influence of punch velocity, blank thickness, and holder force on plastic strain distribution during the deep drawing process as shown in Fig. 13. The results show that increasing punch velocity leads to a noticeable reduction in plastic strain, indicating less severe deformation at higher drawing speeds. In contrast, increasing blank thickness causes a gradual rise in plastic strain due to the higher resistance of the material, which requires greater deformation to be drawn. The effect of holder force appears minimal, with only slight variations in plastic strain across the studied range.

The evolution of plastic strain follows a trend similar to that observed for von Mises stress, as confirmed by the numerical results, response plots, and statistical analysis. Plastic strain decreases with increasing punch velocity, indicating improved deformation stability at higher forming speeds. In contrast, plastic strain increases progressively with blank thickness, whereas blank holder force shows only a minor reducing effect within the investigated range. These findings suggest that punch velocity and blank thickness primarily govern the plastic deformation behavior, while the influence of holder force remains limited.

This response can be attributed to the reduced strain localization at higher punch velocities, which promotes more uniform material flow and lowers the accumulated plastic strain. The increase in plastic strain with blank thickness is associated with higher deformation resistance, leading to greater strain development in the cup wall. Meanwhile, the minimal influence of blank holder force indicates that flange constraint was adequate under the studied conditions, resulting in only slight changes in plastic strain.

## Conclusions

In this study, a comprehensive numerical investigation was conducted to evaluate the influence of punch velocity, blank thickness, and blank holder force on the deep drawing performance of aluminum circular blanks. The analysis focused on maximum thinning, maximum forming force, von Mises stress, effective plastic strain, and Forming Limit Diagram (FLD) behavior. Statistical evaluation using ANOVA within the Response Surface Methodology framework enabled the development of predictive relationships and identification of statistically significant factors and interactions. The principal findings are summarized as follows:

- Punch velocity emerged as the dominant factor, consistently reduced maximum thinning, forming force, stress, and plastic strain, while shifting strain states further below the forming limit, enhancing deformation stability and reducing forming severity.
- Blank thickness increased thinning, forming force, stress, and plastic strain, moving strain states closer to the forming limit and intensifying deformation severity and loading conditions.
- Blank holder force slightly decreased thinning, had minimal impact on forming force, stress, and plastic strain, but influenced strain distribution and flange restraint through interactions.
- Statistical validation using ANOVA and Response Surface Methodology confirmed the trends observed in the response curves.
- Model reliability was high (R² = 0.83–0.89) with strong agreement with adjusted R² values; predicted R² values (0.39–0.54) were acceptable. Adequate Precision (> 4) and low coefficients of variation indicated a robust signal-to-noise ratio.
- Predicted vs. Actual plots showed close alignment, validating the models’ ability to accurately capture parameter–response relationships.
- The mechanics analysis was provided as evidence of process parameter effects on the measured responses, demonstrating both practical and statistical relevance.

This integrated approach enables the prediction and optimization of deformation severity, forming loads, and strain states by simultaneously evaluating maximum thinning, forming force, stress evolution, plastic strain, and forming limit behavior under the coupled influence of the input parameters. It provides practical guidance for industrial aluminum cup drawing. By reducing thinning, limiting peak forming forces, and maintaining safe strain levels, the methodology supports improved dimensional accuracy, tool life, and process efficiency in high-volume manufacturing.

## Data Availability

The raw experimental data used in this study are available from the corresponding author upon reasonable request.
